# Ketamine: Navigating Risk and Benefit. the Margaret Slack Travelling Fellowship

**DOI:** 10.1192/bjo.2026.11167

**Published:** 2026-06-30

**Authors:** Rebecca McKnight, Shanthi Sarma

**Affiliations:** 1 Greater Manchester Mental Health NHS Foundation Trust, Manchester, United Kingdom; 2 Manchester Metropolitan University, Manchester, United Kingdom; 3 Gold Coast Health, Gold Coast, Australia; 4 Bond University, Gold Coast, Australia

## Abstract

**Aims:**

Treatment-resistant depression (TRD) is common and associated with substantial disability and economic burden. It is estimated to affect over 100 million people worldwide and approximately 2.7 million people in the United Kingdom. Ketamine has emerged as a novel therapy for TRD, with studies demonstrating rapid antidepressant effects. However, ketamine is also used recreationally and can result in multi-systemic morbidity, accidental injury, assault and death. In the past decade there has been a marked increase in people entering treatment for harmful ketamine use in the UK, alongside a significant rise in ketamine-related deaths. The Royal College of Psychiatrists Margaret Slack Travelling Fellowship aims to broaden Higher Trainee’s academic knowledge and experience in a centre of excellence.

**Methods:**

Gold Coast Health’s (GCH) Specialist Neurostimulation and Mood Disorder Service is Queensland’s first public service, and one of Australia’s first, to establish a ketamine clinic for TRD. The service is the Queensland site for the TREK trial, a multi-centre randomised controlled trial comparing intranasal esketamine and subcutaneous racemic ketamine for TRD. The service has supported several other multi-centre studies on ketamine that have shown efficacy and safety.

The author was awarded the 2025 Margaret Slack Travelling Fellowship to visit GCH in August 2025, to observe the ketamine clinic, TREK trial, Alcohol and Other Drugs Service, and speak with multi-disciplinary staff and patients to develop clinical insights.

The author attended presentations by Prof Colleen Loo, Black Dog Institute, on “*Extending Knowledge and Clinical Experience on the Use of Ketamine*” and “*Ketamine for the Treatment of Depression in Older Adults*” by Associate Prof, Shanthi Sarma, GCH (11 and 15 August 2025).

**Results:**

The author delivered oral presentations at GCH, “*Understanding Ketamine Use Disorder: Perspectives from England and Considerations for Ketamine Clinics*”, and “*Promoting Awareness on Ketamine Use Disorder: Enabling early identification, treatment and referral*”, at the International Medicine in Addiction conference in Sydney (18 and 30 August 2025).

**Conclusion:**

There is demonstrated interest from British and Australian clinicians in the use of ketamine to effectively and safely treat TRD, in addition to ketamine use disorder, its risks and systemic harms. This interest creates opportunities to strengthen collaboration and shared learning between psychiatry, urology, and addiction disciplines internationally. It also supports the development of improved services for people receiving ketamine for TRD and for those using non-prescribed ketamine, promoting safe, evidence-informed practice.

